# Phenotypes and Karyotypes of Human Malignant Mesothelioma Cell Lines

**DOI:** 10.1371/journal.pone.0058132

**Published:** 2013-03-14

**Authors:** Vandana Relan, Leanne Morrison, Kylie Parsonson, Belinda E. Clarke, Edwina E. Duhig, Morgan N. Windsor, Kevin S. Matar, Rishendran Naidoo, Linda Passmore, Elizabeth McCaul, Deborah Courtney, Ian A. Yang, Kwun M. Fong, Rayleen V. Bowman

**Affiliations:** 1 UQ Thoracic Research Centre, School of Medicine, The University of Queensland, Brisbane, Queensland, Australia; 2 Department of Thoracic Medicine, The Prince Charles Hospital, Brisbane, Queensland, Australia; 3 Department of Anatomical Pathology, The Prince Charles Hospital, Brisbane, Queensland, Australia; 4 Department of Thoracic Surgery, The Prince Charles Hospital, Brisbane, Queensland, Australia; University of Kansas Medical Center, United States of America

## Abstract

**Background:**

Malignant mesothelioma is an aggressive tumour of serosal surfaces most commonly pleura. Characterised cell lines represent a valuable tool to study the biology of mesothelioma. The aim of this study was to develop and biologically characterise six malignant mesothelioma cell lines to evaluate their potential as models of human malignant mesothelioma.

**Methods:**

Five lines were initiated from pleural biopsies, and one from pleural effusion of patients with histologically proven malignant mesothelioma. Mesothelial origin was assessed by standard morphology, Transmission Electron Microscopy (TEM) and immunocytochemistry. Growth characteristics were assayed using population doubling times. Spectral karyotyping was performed to assess chromosomal abnormalities. Authentication of donor specific derivation was undertaken by DNA fingerprinting using a panel of SNPs.

**Results:**

Most of cell lines exhibited spindle cell shape, with some retaining stellate shapes. At passage 2 to 6 all lines stained positively for calretinin and cytokeratin 19, and demonstrated capacity for anchorage-independent growth. At passage 4 to 16, doubling times ranged from 30–72 hours, and on spectral karyotyping all lines exhibited numerical chromosomal abnormalities ranging from 41 to 113. Monosomy of chromosomes 8, 14, 22 or 17 was observed in three lines. One line displayed four different karyotypes at passage 8, but only one karyotype at passage 42, and another displayed polyploidy at passage 40 which was not present at early passages. At passages 5–17, TEM showed characteristic features of mesothelioma ultrastructure in all lines including microvilli and tight intercellular junctions.

**Conclusion:**

These six cell lines exhibit varying cell morphology, a range of doubling times, and show diverse passage-dependent structural chromosomal changes observed in malignant tumours. However they retain characteristic immunocytochemical protein expression profiles of mesothelioma during maintenance in artificial culture systems. These characteristics support their potential as *in vitro* model systems for studying cellular, molecular and genetic aspects of mesothelioma.

## Introduction

Malignant mesothelioma is an aggressive tumour of serosal surfaces (most commonly pleura), usually caused by exposure to asbestos. The incidence is variable worldwide but is highest in Australia: 47 new cases per 100,000 population [1] and UK: 30/1,000,000 per year [2]. Because of long latency from exposure to diagnosis, the incidence of mesothelioma is expected to increase in parts of the world where asbestos was mined or where asbestos products were used. Mesothelioma is resistant to anticancer treatment and median survival from diagnosis remains approximately 10 months [3]. Better understanding of the biology of mesothelioma underpins discovery and implementation of new therapeutic strategies. To this end, *in vitro* models of disease have been important tools for studying biological properties of tumours.

Advantages of cell line models include that they are relatively inexpensive compared with animal models, limitlessly renewable, able to be manipulated by transfections or knockdowns for investigation of gene function, and are practical for high-throughput screening studies. Furthermore, lacking stromal and inflammatory cell content, they provide a relatively pure source of tumour which is superior for estimation of gene copy number. Cultured cells derived from many different tumour types have been shown to retain hallmarks of cancer with the exception of angiogenesis [4], [5].

Mesothelioma cell lines were useful tools to demonstrate that the NF2 tumour suppressor gene product, merlin, exerts its anti-proliferative effect in mesothelioma by suppression of p21 activated kinase-induced cyclin D1 expression [6]. Mesothelioma cell lines have played an important role in understanding the biological role of osteopontin and its isoforms. [7]. *In vitro* screening assays in cell line models can be useful to predict therapeutic responses and mesothelioma cell lines have proved useful models for testing conventional, targeted, and gene-based therapies [8].

Although characterised mesothelioma cell lines exist [9]–[11], few are available in tumour banks such as American Type Culture Collection (ATCC), European Collection of Animal Cell Cultures (ECACC) and Cell Bank Australia. In the era of next generation sequencing, a repository of molecularly and biologically characterised and clinicopathologically annotated early passage cell lines represent a valuable resource for mesothelioma research. However, to work as *in vitro* models of human disease, cell lines must reflect biological and genetic changes of human disease closely and stably.

This study aimed 1) to establish a bio-tool of annotated human malignant mesothelioma tumour cell lines characterised for morphology, doubling times, immunocytochemical properties, and chromosomal alterations, and 2) to define passage related changes in cytogenetic profiles of human mesothelioma cell lines in long term culture.

## Methods

### Generation of Human Malignant Mesothelioma Cell Lines from Clinical Samples

Patients with malignant mesothelioma attending clinics at The Prince Charles Hospital (TPCH) gave written informed consent to donate pleural biopsy tissue and/or pleural fluid after sufficient diagnostic samples were obtained. The study was approved by the Ethics Committee at The Prince Charles Hospital (EC27121). Demographic, clinical and pathology data for the six donors are detailed in Table 1.

**Table 1:** Clinical Annotation of TPCH Cell Lines.

Cell Line	Age	Gender	Histologic type	Origin of Specimen
MM04	86	M	Desmoplastic	Pleural Biopsy
MM05	70	M	Biphasic	Pleural Biopsy
MM12	63	M	Biphasic	Pleural Biopsy
MM13	59	M	Biphasic	Pleural Biopsy
MM1081	69	M	Epithelioid	Pleural Biopsy
PF1038	67	M	Unclassified	Pleural Fluid

Methods to generate cell lines were as follows:

1. Tissue samples from video assisted thoracoscopic (VAT) biopsies were cut into small pieces (approximately 1–3 mm) and plated in sterile petri dishes containing 4 ml medium. Fresh medium (R-10) was added periodically until adherent cell outgrowth was observed. To prepare cell lines, tissue pieces were removed and cells were harvested to fresh flasks and allowed to grow to confluence. Medium was changed every three days and subculturing was performed when the cells became 70–80% confluent.
2. Pleural fluid samples were centrifuged (1766 rpm for 7 min) and the pellets were washed in phosphate buffered saline (PBS, Australian Chemical Reagents QLD, Australia Cat No 1077) and distributed into 75 cm^2^ flasks (Nunc Denmark, Cat No 156499). Medium (R-10) was changed every three days to remove non-adherent cells. Subculturing was performed by treating cells with 0.25% trypsin-EDTA (Invitrogen, Cat No 25200072) for two min at 37°C at confluence.

All cells were cultured in RPMI 1640 (Gibco Cat No 21870-076) with 10% FCS (Lonza, Cat No 14-401F) supplemented with 1% Penicillin/Streptomycin/Glutamine (Gibco Cat No 10378-016) and 0.2% fungizone (Invitrogen Cat No 04195780D) (R-10) and maintained at 37°C in a humidified atmosphere at 5% CO_2_. Early passage cells were stored in liquid nitrogen in freezing medium (90% FCS and 10% DMSO).

### Mycoplasma Testing

All cell lines tested negative for mycoplasma infection using a conventional PCR based Mycoplasma detection kit, Venor GEM (Minerva biolabs) (data not shown).

### Morphological Characterisation

Six cell lines were selected for characterisation. Clinical and demographic details for the six donors and clinical pathology of the primary mesothelioma tumours are shown in Table 1. Histology subtypes were classified as epithelioid in one case, biphasic in three, desmoplastic in one, and undetermined in one case.

Cells were grown over cover slips to 70% confluence, washed and fixed in gluteraldehyde buffer at 4°C for 1 hour and then examined by light microscopy. Transmission electron microscopy (TEM) was used for ultra-structural characterisation. Cells at 70–80% confluency were washed with 1×PBS and fixed with 2 ml 3% gluteraldehyde buffer at 4°C for 1 hr. Cells were harvested with a rubber scraper and spun at 1200 rpm for 5 min. Supernatant was discarded and fresh gluteraldehyde buffer was added. Cell pellets were secondarily fixed with osmium tetroxide and stained *en bloc* with uranyl acetate and embedded in Procure 812 Epoxy Resin. Ultrathin sections were cut at 70–90 nm, stained with lead citrate, and examined by transmission electron microscope (JEOL JEM-1011, 100 kV, JEOL Australasia Pty Ltd).

### DNA Fingerprinting

To verify derivation, cell line profiles for 45 SNPs generated by Sequenom using the iPLEX Sample ID Plus Panel were compared to peripheral constitutional DNA SNP profiles from each donor.

### Growth Characteristics

5000 cells of each line at passage number 8–16 were seeded into triplicate wells of a 24 well plate (2500 cells/cm^2^) and grown in medium (R-10). Cells were counted after 24 hr, 48 hr, 72 hr, 96 hrs, 7 days, 9 days and 11 days. Cell viability was assessed by trypan blue exclusion. Doubling time was calculated by the algorithm provided by http://www.doubling-time.com. [12].

### Cytogenetic Analysis

Chromosome preparations of the cells were made as follows: Colcemid (Demecolcine Solution D1925 Sigma 10 ug/ml in HBSS) (0.04 ug/ml final concentration) was added to semi- confluent cultures (70%) 16–17 hours before harvesting. Metaphase cells were harvested by trypsinizing, treated with a hypotonic solution (0.56% KCl) for 15 min at 37°C, and fixed twice in acetic acid (one part)/methanol (three parts). Cells were dropped onto clean slides and sent to Applied Spectral Imaging (Israel) for Spectral Karyotyping (SKY). A SKY probe containing all 24 labelled chromosome libraries was hybridized simultaneously to the metaphases. After washing_,_ slides were stained with 4′6–diamidino-2 phenylindole (DAPI) in antifade medium. Selected metaphases were captured and analysed using the SD 300 bioimaging system (ASI Ltd), using manufacturer’s software for acquisition and analysis of colour chromosomes (HiSKY). DAPI images were captured separately and chromosomes were then sorted automatically into a karyotype table and analysed for structural and/or numerical aberrations. From each sample 7–10 metaphases were randomly chosen for full analysis from different areas of the slides.

### Immunocytochemical Studies

Staining indicative of mesothelial origin was performed using the following antibodies: anti-calretinin (Zymed Invitrogen, Carlsbad CA Cat No 180211) at a dilution of 1∶50, and anti-cytokeratin-19 (Dako, Clone RCK108) at a dilution of 1∶100 for staining cells. Cells were grown on cover slips to 80% confluency. Staining with immunocytochemical markers was performed using streptavidin–biotin horseradish peroxidase (SAB/HRP) kit (Dako, North America, Carpentaria, CA, Cat No K3468) with an incubation time of 1 hour at room temperature for each primary antibody. Intensity of immunocytochemical reactions was evaluated by a semi-quantitative method using a scale defined as follows: negative: no positive cells, weak: <10%, medium: 10–60%, high: >60%. Cell line JU77, a previously well-characterised malignant mesothelioma cell line [9], was used as a positive control, and cells stained without primary antibody were taken as negative controls.

### Anchorage-independent Growth

Anchorage-independent growth strongly correlates with tumourogenicity [13]. Soft agar colony formation assays were performed to assess this property. Briefly, 0.5% agar (base layer) and 0.37% agar (top layer) (Sea Kem agar, FMC, Philadelphia, PA) was prepared. The base layer was poured into a 96-well cell culture plate and allowed to set for 5 minutes. The top layer was mixed with 10000 cells and poured over the base layer. Cells were allowed to grow for 10 days until colonies were visible. WST-1 was added to each well, incubated at 37°C, and plates were read at optical density at 450 nm [14]. The A549 (human lung adenocarcinoma cell line) cell line was used as a positive control and neonatal foreskin fibroblasts as a negative control in this assay.

## Results

Viable cells from all lines reported here were retrievable from cryopreservation, subsequently grew to confluency, and were propagated by passaging. DNA fingerprinting was undertaken on four of the cell lines showing matching SNP profiles between blood and cell line DNA (MM04, MM05, MM13, MM1081) (data not shown) verifying derivation of each line from the donor thus authenticating the annotation of these lines against clinicopathologic characteristics of the donor patients and their tumours.

### Morphological, Ultrastructural, and Immunohistochemical Properties of Cultured Human Mesothelioma Cells

**Figure 1:**
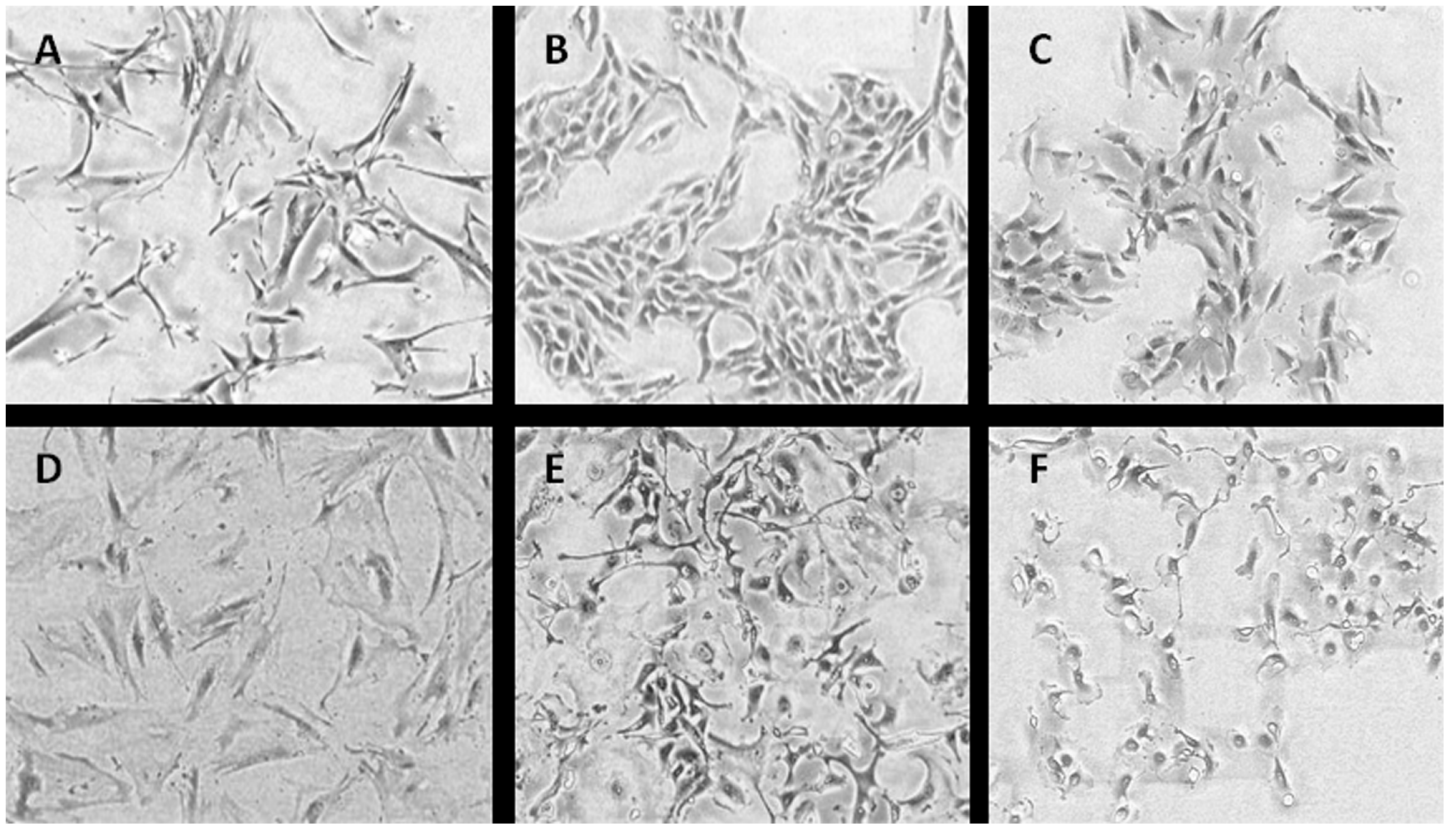
Light microscopy of cell lines. Figures showing morphology using light microscopy MM04 (A), MM05 (B), MM12 (C), MM13 (D), MM1081 (E) and PF1038 (F).

**Figure 2:**
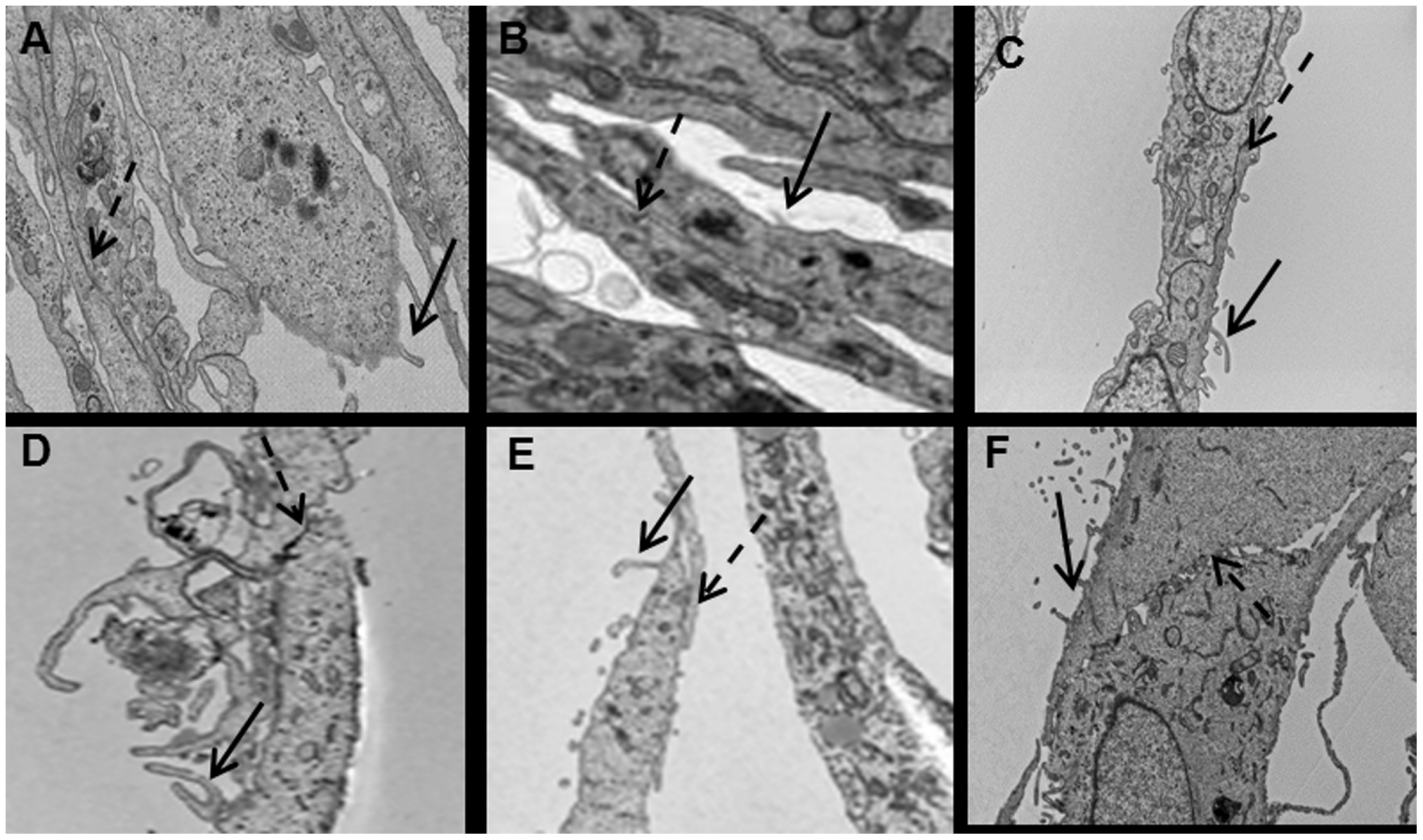
Transmission electron micrograph of cell lines. Cell lines showing microvilli (dark arrows) and tight intracellular junctions (dotted arrows). MM05 (A), MM04 (B), MM12 (C), MM13 (D), MM1081 (E) and PF1038 (F).

All cell lines (MM04, MM05, MM12, MM13, MM1081 and PF1038) formed monolayers with various morphological cell types (Figure 1, Table 2). PF1038 displayed two different morphologies in early passages (p6) (spindle shaped and rounded cells), but spindle cells were dominant in later passages (p16) (Figure 1). Transmission electron microscopy in all six lines showed characteristic features of mesothelioma ultrastructure: slender short microvilli lacking an underlying terminal filamentous web, cytoplasm containing organelles and intermediate filaments, and tight intercellular junctions (Figure 2) [15]. Cells from all six lines [MM04 (p4), MM05 (p5), MM12 (p5), MM13 (p2), MM1081 (p5) and PF1038 (p3)] stained positively for calretinin and cytokeratin 19 by immunocytochemistry (Table 2).

**Table 2:** Morphological, growth and immunocytochemical characteristics of cell lines.

Cell Line	Morphology	Doubling time (hrs)	Immunocytochemistry	
			Calretinin	Cytokeratin 19
MM04	Spindle shaped cells with few vacuoles	63.74 hr	High	High
MM05	Spindle shaped cells with few vacuoles	54.51 hr	Weak	High
MM12	Cells with Irregular membranes with thick processes.	37 hr	Medium	Medium
MM13	Thick stellate shaped cells	72 hr	Medium	Medium
MM1081	Thick stellate shaped cells	48.8 hr	High	Medium
PF1038	Mixed cell morphology some round with irregular membranes and some spindle shaped cells	44 hr	High	High

### Growth Characteristics

**Figure 3:**
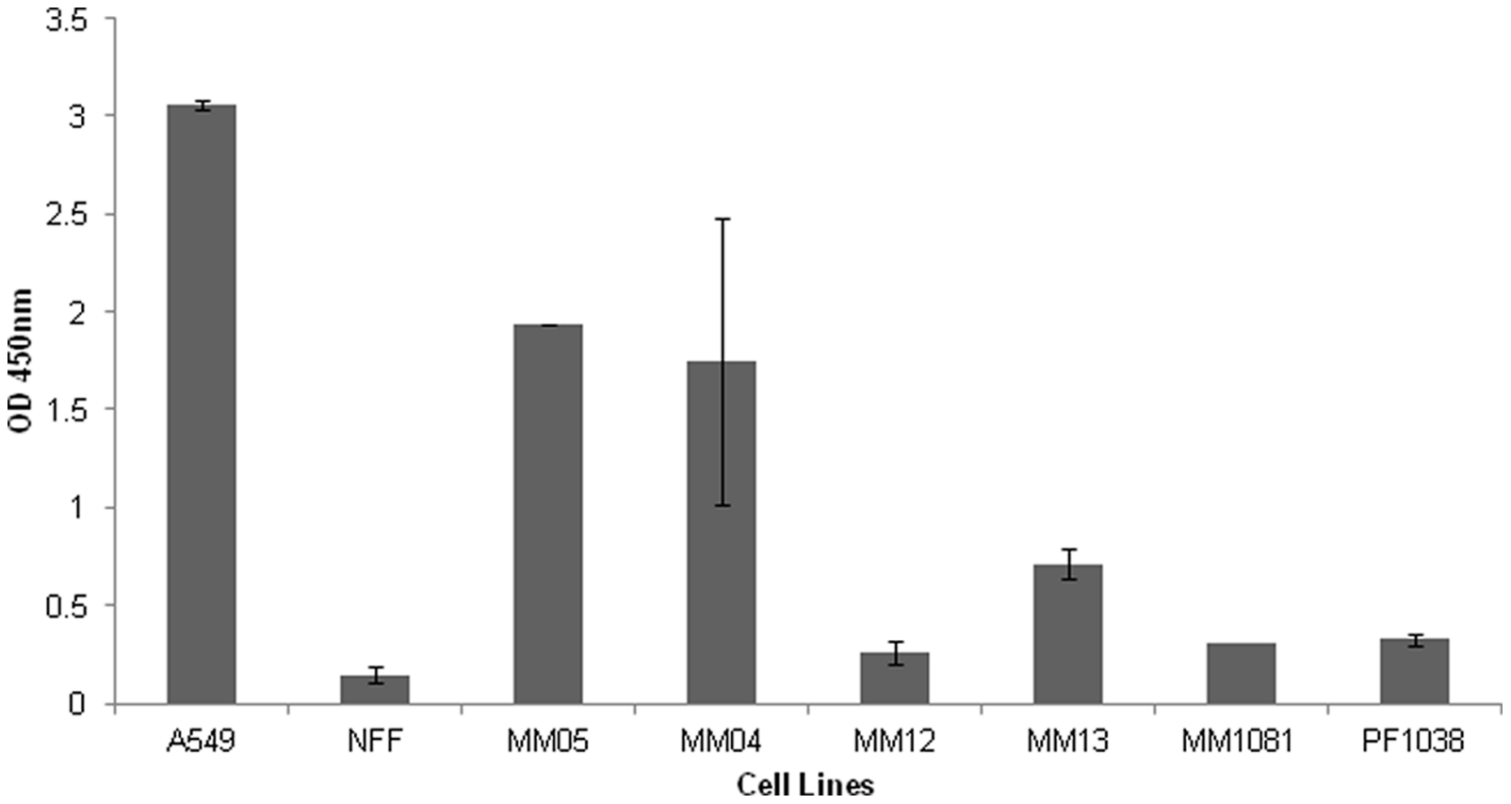
Anchorage independent growth assay. The assay showing the colony formation for all the cell lines (A549, NFF, MM04, MM05, MM12, MM13, MM1081 and PF1038). 10,000 cells were grown on 96 well plates on soft agar for 10 days before adding Wst-1. Optical Density (OD) was measured at 450 nm and referenced at 620 nm. A549 (Human lung adenocarcinoma epithelial cell line) cell line was used as a positive control and NFF (Neonatal foreskin fibroblasts) as a negative control.

Growth characteristics were studied at the following passages (p) - MM04 (p11) MM05 (p16), MM12 (p5), MM13 (p9), MM1081 (p6) and PF1038 (p6). Doubling times ranged between 30 hr and 72 hrs (Table 2). No relationship was found between doubling times and histological subtype classification. All lines formed colonies in soft agar, consistent with anchorage-independent growth capacity (Figure 3).

### Karyotypes

Chromosomal abnormalities are listed in Table 3. MM12 p10 (biphasic) and MM1081 p4 (epithelioid) both showed polyploidy. A single copy of chromosomes 8, 14 and 22 was observed in two cell lines (MM04 p8 and PF1038 p12) and of chromosome 17 in MM04, MM05p5 and PF1038. Deletions and translocations were commonly seen in chromosomes 1, 5 and 6 in epithelioid cell line (MM1081), and in chromosomes 1, 9, and 19 in biphasic cell lines (MM05 and MM12). In MM04 four different karyotypes were observed at passage 5. One cell line (MM13) could not be karyotyped due to condensed metaphase spreads.

**Table 3:** Karyotypic analysis of cell lines.

				Chromosome number																							
Cell Line		n	N	1		2				3		4		5		6		7			8	9			10		11
MM04 P8		7	45	der(1) t (1;15)								der(4) t (4;5)		del 5													
			45	der(1) t (1;15)								der(4) t (2;4)		del 5													
			45	der(1) t (1;17)								del(4)X2		del 5							−8						11
			44	der(1) t (1;17)								del 4		−5				7			der(8) t (8;17)						11
MM05 P5		8	41	der(1) t (1;17)								del 4									der(8) t (8;17)	der(9) t(9;15)					t (11;18)
																						−9					
MM12 P10		10	95–96	der(1) t (1;7;12)		der(2)t(2;19)				der(3)t(X;3)								der(7)t(1;7)				der(9) t(9;11)					
										der(3)t(1;3)								der(7)t(7;13)									
										der(3)t(3;4)																	
										der(3)t(1;3;4)																	
MM1081 P4		7	59–113	der(1) t (1;19)		der(2)t(2;6)								del 5		der(6) t (1;6)					der(8) t (8;9)	der(9) t(9;16)					
PF1038 P12		8	42–45	der(1) t (1;17)		der(2)t(2;8)															−8						−11
																					der(8) t (8;9;8)	der(9) t (2;9;8)			der(10) t (8;10)		der(11) t (11;14)
					**Chromosome number**																						
**Cell Line**	**n**		**N**		**12**		**13**	**14**	**15**		**16**		**17**		**18**		**19**		**20**	**21**			**22**	**X**		**Y**	
MM04 P8	7		45					−14			der(16) t (5;16)												−22				
											der(16) t (14;16)																
			45					−14															−22				
			45					−14			der(16) t (14;16)		−17										−22				
			44					−14			−16		−17										−22				
MM05 P5	8		41					−14	−15				−17				−19										
MM12 P10	10		95–96								der(16) t (16;21)						der(19) t (2;19)			der(21) t (16;21)				2X		der(Y) t (Y;10)	
																								der(X) t (X;3)			
MM1081 P4	7		59–113										der(17) t (11;20)											2X		der(Y) t (Y;15)	
PF1038 P12	8		42–45		del(12)			−14					−17										−22	−X			
					der(12) t (5;12)																						

n = Total number of metaphases studied, N = Total number of chromosomes.

### Passage-related Changes in Karyotype

Two cell lines (MM04 and MM05) studied at both early (p8 and p5 respectively) and late (p40 and p42 respectively) passages displayed chromosomal abnormalities. MM05 developed polyploidy at late passage while continuing to show similar abnormalities as observed in early passages. Late passages showed similar translocations in chromosome 1, 8, 11 and 17 as in early passages (Table 4). Early passages of MM04 had shown four distinct karyotypes whereas only one was observed at late passage (Table 4).

**Table 4:** Spectral Karyotypes for Early and Late passages for MM04 and MM05 cell lines.

Cell Line	Karyotype
MM04 (P8)	1/45,XY, −14, −22, +del(5), der(1)t(1;15),t(4)t(4; 5), der(16)t(5;16), der(16)t(14;16)
	1/45,XY, −14, −22, +del(5), der(1)t(1;15),t(4)t(2;4)
	1/45,XY, −14, −22, −17, −8, +11,+del(5), der(1)t(1;17),del(4)X2, der(16)t(14;16)
	1/44,XY, +11, +7, −14, −22, −17, −5, −16,+der(8)t(8;17)der(1)t(1;17),del(4), der(8)t(8;17)
MM04 (P40)	45–46,XY, −14, −22, +del(5), +der(16)t(5;16), der(1)t(1;15), t(3;16), der(4)t(1;4), del(8),der(16)t(14;16)
MM05 (P5)	41, XY, −9, −14, −15, −17, −19, der (1) t (17), del (4), 2Xder (8) t (8;17), 2X der (11) t (11;18) and der (18) t (11;18)
MM05 (P42)	84, XXYY, same translocations

## Discussion

Establishment of primary cell lines as a disease model is potentially of considerable importance in the study of mesothelioma at cellular, molecular and genetic levels. Mesothelioma is readily propagated in cell culture from human tumour biopsies and pleural effusions. Over a period of four years (2008–2011), we established twenty-one cell lines (defined as successful subculture from a primary culture [16]) from twenty-five samples (fifteen resected human tumour biopsies and ten pleural effusions), representing an overall success rate of 84%. As models of the human disease these cell lines can be studied and manipulated to answer specific research questions, and potentially offer researchers with specific goals access to systems which closely parallel human disease. To assess this potential we describe here the biological characteristics of six of these newly established cell lines.

### Morphology and Growth Characteristics

The morphological and growth characteristics of the six cell lines reported here were consistent with those previously documented for mesothelioma cell lines [9]–[11]. One cell line (PF1038) underwent morphological change during passaging possibly as a result of selective adaptation to artificial culture conditions [17]. Microvilli are characteristic of mesothelial cells. TEM ultrastructure of cultured mesothelioma cells showed microvilli of various lengths in different cell lines, but overall the microvilli in cultured mesothelioma cells were shorter than those observed in histological samples of mesothelioma. Variable length of microvilli in mesothelioma cell lines was also reported by Pass et al [18]. Cell doubling time is an important parameter for both drug screening and functional studies, as the outcome of these types of experiments could be affected by cell cycle phase. Doubling times for early passages of these lines varied between 30 and 72 hours, within the range of doubling times previously reported [9], [18], [19]. All of these mesothelioma lines exhibited anchorage independent growth (colony forming capacity in semisolid media), a hallmark of malignancy [20], the most accurate and stringent *in vitro* assay for tumourigenic potential, and highly correlated with tumourigenicity in animals [13], [21]. We found these cell lines suitable both as plastic adherent cultures and in soft agar for testing the activity of existing chemotherapeutic agents (results to be published separately).

### Immunocytochemistry

There is currently no individual immunohistochemical marker that provides 100% specificity and high sensitivity for diagnosing mesothelioma, nor any marker with 100% negative predictive value. The most useful mesothelial and epithelial markers proposed for the diagnosis of mesothelioma are calretinin (a vitamin D-dependent calcium-binding protein involved in calcium signalling), HBME-1, thrombomodulin, WT-1, mesothelin, and podoplanin as mesothelial markers and pCEA, Ber-Ep4, TTF-2, B72.3 as epithelial markers [22]. High molecular weight cytokeratins (18&19) are expressed in mesothelial cells [23] and soluble cytokeratin-19 was previously found in high concentration in two familial cases of mesothelioma [24]. The cytokeratin-19/CEA ratio is a useful diagnostic marker for malignant mesothelioma [25]. Although an extended panel of IHC markers would enable more comprehensive characterisation, all six cell lines expressed cytokeratin-19 and calretinin.

### Cytogenetic Alterations

Mesothelioma cell lines exhibit complex structural and numerical chromosomal abnormalities on spectral karyotypic analysis - a molecular cytogenetic technique by which even complex chromosomal abnormalities can be detected, which may not be revealed by routine G banding or FISH techniques. Monosomy 17, found in three of five cell lines, has been reported previously in mesothelioma [26], and may be implicated in functional inactivation of the p53 gene due to binding of SV40 Tag protein [27]. Rearrangements involving chromosome 1 were found in all mesothelioma cell lines. Breakpoints and deletions at chromosome 1 regions located near Blym, L-Myc and sci proto-oncogenes have been described in various solid tumours [28] including mesothelioma [29]. Multiple regions of allelic loss from 6q have been reported in mesothelioma [30], [31], and we observed chromosome 6 rearrangements in one (of five) line. Allelic losses at three known tumour suppressor regions (22q which includes Nf2 marker (NF2CA3), 9p for the p16 gene, and 3p for FHIT gene) and at other areas of 14q and 6q, are also frequent in mesothelioma [32]–[34]. On CGH, Lindholm and coworkers found losses in regions of 1p, 3p, 6q, 9p, 13, 14 & 22 and gains in 17q [35]. Both partial loss and total monosomy of chromosome 22 have been observed in mesothelioma previously [31], [36], [37], and we observed monosomy 22 in two of the five lines described here. [35].

### Passage-related Changes

Genomic instability is recognized to occur during long term culturing of tumour cell lines [33]. Polyploidy, which we observed in one cell line (MM05), is reported as a frequent occurrence during long term culturing [38]. Zanazzi and colleagues also reported passage related changes in a mesothelioma cell line, noting disappearance of certain copy number alterations in later passages and appearance of new aberrations [39]. We also found evidence of selection, with reduction to one karyotype in later passages of MM04 suggesting selection of a subpopulation of cells with this karyotype; which perhaps conferred some type of growth advantage.

Mesothelioma lines available from various cell banks may be affected by culture related cytogenetic and other alterations affecting their suitability for various research purposes. Annotation of repository cell lines with evidence of authenticated derivation, passage number, and comprehensive characterisation is essential to underpin their utility as *in vitro* model systems of human disease. Characterization of these six cell lines adds to the bioresources available for future mesothelioma research.
